# Myelodysplastic Syndrome: Riding the Crest of the Wave

**DOI:** 10.3390/jcm11061606

**Published:** 2022-03-14

**Authors:** Fernando Ramos

**Affiliations:** Department of Hematology, Hospital Universitario de León, Altos de Nava s/n, E-24071 León, Spain; mail@fernandoramosmd.es

Myelodysplastic syndrome (MDS) is a group of clonal disorders that arise in pluripotent bone marrow stem cells and present with characteristic phenotypical features (both morphological and flow cytometrical), as well as genotypical abnormalities. Bone marrow failure in the face of a non-hypocellular bone marrow, and in the absence of myelophthisis or hypersplenism, (a situation called “ineffective hemopoiesis”), is the functional hallmark of the disease and morphological dysplasia (“dyshemopoiesis”) is its morphological correlate. This predominantly morphological diagnosis and the prior absence of a definite treatment strategy to modify their natural history contributed to the predominantly conservative attitude that surrounded MDS management in earlier times. More recently, the growing evidence on the neoplastic nature of the disease (molecular changes are observed in the vast majority of MDS patients and shared with other types of cancer), have brought MDS over to the cancer field. These genetic changes confer a genomic instability that contributes to progressive bone marrow failure and may evolve some 20–30% of MDS cases into acute myeloid leukemia (AML), claiming for a more pro-active management. Older alternative catch-all terms such as “refractory anemia” or “preleukemia” are nowadays held inappropriate for this heterogenous group of neoplastic disorders, which are considered different stages of myeloid cancer by the World Health Organization. In this *Special Issue on Diagnostics and Management of Myelodysplastic Syndrome*, we intend to update the core issues of MDS with the participation of prominent hematologists from Spain and Italy.

MDS diagnosis was, and still is, mainly morphological, but modern cytogenetics and next generation sequencing (NGS) are now powerful tools that help us demonstrate the clonal nature of the disease in doubtful cases. NGS has also allowed us to go several steps backward in the natural history of the disease, and it has been instrumental to discover that MDS commonly evolve from a previous mutational stage known as “clonal hematopoiesis of indeterminate potential” (CHIP), whose prevalence is largely age-dependent and characterized by the presence of mutations in a limited number of driver genes, in the absence of cytopenia. In recent years, CHIP has captured the attention of many physicians because of its apparent association to cardiovascular disease [1,2], including ischemic heart disease, peripheral vascular disease, cardiac failure and calcified aortic stenosis. It is also a growing concern for those involved in the treatment of solid tumors and hematopoietic cell transplantation, because the presence of CHIP at diagnosis may increase the risk of therapy-related myeloid neoplasms several years after receiving either standard-dose or high-dose chemotherapy. By contrast, the use of allogeneic donors harboring CHIP might be associated with a reduced relapse rate and a better patient survival, due to the modulation of the subtle balance between graft-versus-host disease and the graft-versus-leukemia effect [3].

MDS pathogenesis is quite complex, but increased apoptosis of bone marrow precursors has long been considered the hallmark of the disease and the underlying mechanism of the dyshemopoietic features observed in patients’ peripheral blood and/or bone marrow. Nowadays, both pyroptosis (caspase-1 mediated, NLRP3 inflammasome-induced cell death) and, to a lesser extent, apoptosis (non-inflammatory cell death), are considered the main pathophysiological mechanisms involved in MDS ineffective hemopoiesis. Autoimmune and auto-inflammatory diseases, including the recently described severe adult-onset inflammatory syndrome (VEXAS) [4], frequently associate to MDS, may precede or follow MDS diagnosis, and pyroptosis has become the main link between these diseases. Inflammasome activation contributes to the initial genotoxicity of MDS cases, as well as to the evolving genomic instability and the final transformation into AML.

The current standard for prognostication of most patients diagnosed with MDS is the Revised International Prognostic Scoring System (IPSS-R). By contrast to the earlier IPSS, in IPSS-R the prognostic weight of the cytogenetic abnormalities is higher than that of marrow blast proportion, and the number and depth of peripheral blood cytopenia is scored in detail. A significant clinical advance has been the ability to ascribe to the “lower risk” vs. “higher risk” group the patients formerly allocated to the ambiguous IPSS-R “intermediate” group by using a straightforward 3.5 score point cut-off. Those patients included in the lower-risk subset may benefit from additional stratification by the MD Anderson Lower-risk Prognostic Scoring System (LR-PSS) that shows a better performance than IPSS-R in this population. Cheng-Liang (Department of Hematology, Hospital Universitario Morales Meseguer, Murcia, Spain) offers us a guided review to current MDS prognostication, including the independent contribution of mutational studies [5].

Therapy of MDS patients ranges from clinical observation to hematopoietic cell transplantation, depending on IPSS-R or LR-PSS scores, mutational findings, as well as age and general condition (including, but not limited to, comorbidity and performance status). Some breakthrough advances in this field have been the widely used erythropoietic stimulating agents (erythropoetin or darbepoetin), lenalidomide therapy for those MDS patients with a low IPSS-R score and “isolated” abnormalities in the long arm of chromosome 5 (commonly known as “5q- syndrome”) and hypomethylating agent therapy (mainly azacitidine) for higher risk patients. New erythropoietic agents (lustapercept) and apoptosis modulators (imetelstat, venetoclax, eprenetapopt), to name just a few, are knocking at our door, and some of them, either alone or in combination, are very promising agents for MDS patients. The progressive addition of patient general condition evaluation to the stratification and management of MDS patients, coupled to the progressive use of NGS and targeted therapy, is paving the way to the much desired “personalized precision medicine” [6,7], in MDS patients. Palacios-Berrquero and Alfonso-Piérola (Department of Hematology, Clínica Universidad de Navarra, Pamplona, Spain) have summarized current MDS therapy in this Special Issue [8].

Lastly, MDS shows some degree of overlap with aplastic anemia and paroxysmal nocturnal hemoglobinuria (PNH), but they are distinct clinico-pathological entities. In order to help delineate them and as a supplementary service to *JCM* readers, we have included at the end of this Special Issue an interesting and patient-oriented short review on PNH, contributed by Fatizzo et al. (Fondazione IRCCS, Milan, Italy) [9].
